# Diabetes Promotes DMH-Induced Colorectal Cancer by Increasing the Activity of Glycolytic Enzymes in Rats

**DOI:** 10.1371/journal.pone.0110455

**Published:** 2014-10-17

**Authors:** Yanglei Jia, Gang Xu, Wenjing Zhou, Zhenzheng Wang, Linlin Meng, Songnan Zhou, Xia Xu, Huiqing Yuan, Keli Tian

**Affiliations:** 1 Department of Biochemistry and Molecular Biology, Shandong University School of Medicine, Jinan, Shandong, China; 2 Department of Gastroenterology, 456 Hospital of PLA, Jinan, Shandong, China; 3 Department of Pathology, 456 Hospital of PLA, Jinan, Shandong, China; Southern Illinois University School of Medicine, United States of America

## Abstract

The objective of the present study was to investigate the association between diabetes mellitus and colorectal carcinogenesis as well as the possible mechanism involved in this interaction. Diabetes rat models were induced with a low dose of STZ followed by a low dose of DMH to induce colorectal cancer. The formation of ACF in the colon and the incidence, number and size of tumors were measured. The activity of glycolytic enzymes in colonic tissues was also measured. The results demonstrated that both the total number of ACF and the number of foci that contain a different number of crypts were increased in diabetic rats. At the end of the experimental treatment, the incidence, number and size of tumors were also increased in diabetic rats. Overall, these data indicated that diabetes increased the risk of colorectal cancer. The activity of HK and PK in colonic tissues was increased in diabetic rats, whereas the activity of PDH was decreased. In addition, the activities of these enzymes in intratumor were higher than that of in peritumor. These data indicated that the high rate of glycolysis may play a role in colorectal carcinogenesis in diabetic rats.

## Introduction

Colorectal cancer is a common gastrointestinal malignant tumors. It is the third most common cancer and the third leading cause of cancer death in men and women in the United States [1], [2]. In recent years, with the improvement of living standards and lifestyle changes, the incidence of colorectal cancer has increased year by year in developing countries. Several similar risk factors have been found between diabetes mellitus and colorectal cancer. Those include a “Western Lifestyle”, with diets low in fruits and vegetables or fiber and high in fat and cooked meat, as well as restricted physical activity [3], [4]. Epidemiologic findings to date have shown that type 2 diabetes mellitus is closely related to the increased risk of colorectal cancer [3]–[8]. However, some results are inconsistent with this association because diabetes mellitus is a group of complex metabolic disorders characterized by hyperglycemia. Several confounding factors, including duration of diabetes, varying levels of metabolic control, usage of different drugs for therapy, and the possible presence of chronic complications, complicate the accurate assessment of cancer risk in diabetic patients. In addition, different epidemic factors from different countries, ethnicities and regions may influence the association between diabetes and cancer [3],[7],[9].

The possible mechanisms involved in diabetes mellitus-related cancer may be associated with long-term insulin resistance and hyperinsulinemia, which have been extensively described to date [10]–[13]. Hyperinsulinemia may affect the occurrence and development of colorectal cancer through a variety of mechanisms. An abnormal increase of insulin and insulin-like growth factor-1 (IGF-1) in the serum can lead to the occurrence of tumors by promoting the transformation and proliferation of colorectal epithelial cells, influencing the cell cycle and inhibiting cell apoptosis [10]. Patients treated with insulin for a long period of time have a higher risk of cancer, and with the increasing duration of insulin treatment, the incidence of cancer may increase [12],[13]. Nevertheless, it is possible that a particular factor could affect the risk of colon cancer both by influencing the concentrations of insulin and through other mechanisms [14],[15].

In the presence of oxygen, most cells primarily metabolize glucose to pyruvate via glycolysis and then completely oxidize pyruvate to carbon dioxide through the citric acid cycle. However, tumor cells generate as much as 60% of their ATP through glycolysis, and the glycolytic rate of tumor cells is up to 200 times higher than that of normal cells, regardless of the presence or absence of oxygen [16],[17]. This phenomenon, recognized approximately seven decades ago, is known as the Warburg effect. This phenomenon is caused by the abnormal expression of many glycolytic enzymes. Several key enzymes, such as hexokinase (HK) and pyruvate kinase (PK) are known to play a crucial role in initiating and maintaining the high rates of glucose catabolism of rapidly growing tumors [18],[19]. PK has also been reported to contribute to the accumulation of intermediates of glycolysis for the following anabolic processes: the synthesis of nucleic acids, amino acids, and phospholipids [20]. PK imparts a growth advantage to tumor cells, particularly under hypoxic conditions.

The main features of diabetes mellitus are hyperglycemia and the dysfunction of glucose metabolism. Due to hyperglycemia, the permeability of capillary vessels is decreased and the mitochondrial enzymes that are related to glucose metabolism were inhibited [21], therefore the carbon metabolism of oxidative phosphorylation is inhibited. In diabetic patients, normal cells with a low rate of glycolysis cannot obtain enough energy from glucose metabolism and may die. However, cells with a high rate of glycolysis can compensate the damage of enzymes in mitochondria and generate more ATP through glycolysis to maintain the rapid proliferation and transformation of normal cells to tumor cells. Several key enzymes and proteins involved in glucose metabolism in diabetic rats were investigated by Mansor et al. [22]. The activity of PDH, a highly regulated enzyme of mitochondrial glucose metabolism, and glucose transporter 4 (GLUT4) were significantly decreased. The PDH inhibitor pyruvate dehydrogenase kinase 4 (PDK4) level was increased. These results revealed the low rate of oxidative phosphorylation in mitochondria in diabetic rats.

In the present study, diabetes was induced in Sprague–Dawley (SD) rats using STZ, followed by the induction of colorectal cancer using DMH. We sought to determine whether Type 2 diabetes mellitus was associated with the risk of colorectal cancer in rats and the possible mechanisms involved in this process.

## Materials and Methods

### Reagents

STZ, DMH and methylene blue were purchased from Sigma (St. Louis, MO). Commercial kits for immunostaining of PCNA were from Beijing Zhongshan Goldbridge Biotechnology Co. (Beijing, China). The assay kits for detecting the activities of hexokinase (HK) and pyruvate kinase (PK) were purchased from Nanjing JianCheng Bioengineering Institute (Nanjing, China).

### Animals

Sixty male Sprague–Dawley (SD) rats (160–180 g) were purchased from the Vital River Laboratory Animal Technology Co. Ltd (Beijing, China). All rats were housed in standard polypropylene cages (3 rats/cage) and kept in a room under standardized conditions (22±3°C, 12 h light/12 h darkness, humidity 50±10%) with free access to food pellets and tap water. Animals were allowed to acclimate for 1 week on chow and water. The rats were randomly divided into four groups of 15 animals each: (1) the Control group; (2) STZ group; (3) DMH group; and (4) STZ+DMH group. The rats in the control group and DMH group were provided with a normal pellet diet (NPD), and those in the STZ group and STZ+DMH group were provided with a high-fat diet (HFD). The composition and preparation of HFD (Table 1) were as previously described [23]. The body weight of rats was measured once a week. All studies were performed with the approval of the animal experimental ethics review committee of Shandong University School of Medicine.

**Table 1:** The Composition of HFD.

Ingredients	Diet (g/kg)
Powdered NPD	550
Lard	200
Saccharose	200
Yolk	40
Cholesterol	10

### Development of diabetes model

After 2 months of dietary manipulation, the rats in the STZ group and STZ+DMH group were injected intraperitoneally (i.p.) with a low dose of STZ (35 mg/kg body wt; STZ was dissolved in 0.1 mol/L citric acid buffer, pH 4.4), while the rats in the control group and DMH group were given a vehicle citrate buffer (pH 4.4) in a volume of 1 ml/kg, i.p. Blood samples were drawn immediately before and 1 week after injection of STZ or its vehicle from the caudal vein after fasting for 24 hours.

### Development of colorectal cancer

Two weeks after the injection of vehicle or STZ, the rats in the intervention groups were injected (i.p.) with either DMH (25 mg/kg body wt; DMH was dissolved in normal saline) or 0.9% NaCl once a week for 12 weeks. One week after the last injection of DMH, five rats from each group were sacrificed after fasting for 24 hours for ACF identification. Blood samples were collected by cardiac puncture. Colon tissues were collected and divided into two parts. One part was stored in liquid nitrogen to detect the activity of enzymes, and the other part was for the observation of ACF. Twelve weeks after the last injection, the remaining rats were sacrificed for the calculation of tumor incidence, average number of tumors and tumor volume. Then, the colon tissues were harvested and divided into two parts. One part was stored in liquid nitrogen to detect the activity of enzymes, and the other part was fixed in 4% paraformaldehyde for pathology analysis. All of the animals were sacrificed by CO_2_ asphyxiation.

### Blood parameters

Blood samples were collected and centrifuged at 1000 g for 10 min to collect the serum, which was stored at −20°C until analysis. Fasting blood glucose (FBG) levels were measured by the glucose oxidase method (GOD, Applygen Technologies Inc., Beijing, China). The serum concentrations of triglyceride (TG), total cholesterol (TC), low-density lipoprotein cholesterol (LDL-C), and high density lipoprotein cholesterol (HDL-C) were determined using an automated biochemical analyzer (TOSHIBA-40FR). The serum insulin (INS) concentrations were measured using enzyme-linked immunosorbent assay (ELISA) kits according to the manufacturer's protocol (Cusabio, Wuhan Huamei Biotech Co. Ltd., Wuhan, China).

### Identification of ACF

ACF was identified as described previously. Briefly, the colons were harvested and washed with 0.01 mol/L PBS followed by fixation in 4% neutral-buffered paraformaldehyde for 24 h. The colon tissues were stained in 0.5% methylene blue solution for 5 min, followed by one rinsing wash. The total number of ACF and the number of aberrant crypts (ACs) in each focus were counted under a light microscope (40×).

### Calculation of tumor incidence, number and volume

Tumor incidence is the number of rats that tumors were developed in the colon tissue with the repeated injection of DMH during the whole experiment process. The rats that tumors were visualized in general by gross examination in colon tissue at the end of the experiment were indicate tumor-bearing rats. The tumor incidence, average tumor number and tumor size were calculated using the following formulas [24]:
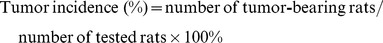


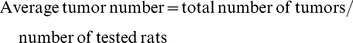


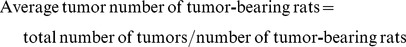


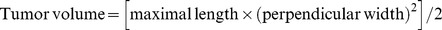



### Histological assay and Proliferating Cell Nuclear Antigen (PCNA) staining

Normal colon tissue, colon tumor and tissues adjacent to tumors were harvested and fixed in 4% neutral-buffered paraformaldehyde for 24 h. The tissues embedded in paraffin were cut into 4-µm sections on histology slides and stained for hematoxylin and eosin (HE) staining and PCNA immunostaining using a streptavidin-biotin-immunoperoxidase complex method (a commercial kit) according to the manufacturer’s instructions. Brown-yellow stained nuclei were considered positive. For determination of the proliferative index, five high power lens (HP) visual fields (200 cells each) consisting of a total of 1000 cells were counted. The proliferative index (PI) was expressed as the percentage of PCNA-positive nuclei among the total number of cells counted.

### Assay of enzyme activity

The enzyme activities were measured in both the peritumoral and tumoral regions. If no tumors were found, the tissue samples were dissected at random. Frozen tissue (0.1 g) was homogenized in a glass homogenizer with 0.9 ml of normal saline. To preserve the activity of enzymes, the entire grinding process was performed on ice. The activity of hexokinase (HK), pyruvate kinase (PK) and pyruvate dehydrogenase (PDH) were measured.

### Statistical Analyses

All results were presented as the means ± S.E.M. and all P values reported are two-tailed and P<0.05 was considered statistically significant. For parameters with Gaussian distribution, comparisons of body weight between each two groups and also between NPD-fed and HFD-fed rats, the ‘number of ACF’, the ‘Tumors/All rats’, ‘Tumors/Tumor bearing rats’ and ‘Volume of Tumors’ between DMH and STZ+DMH group, the enzymes activities between the peritumoral and the intratumoral and also between each two groups were all performed using unpaired student’s T test. Tumor incidence was compared using Chi square test. Data analysis was performed employing the statistical package for social science, version 18 (SPSS Software, SPSS Inc., Chicago, USA).

## Results

### Physical parameters

**Figure 1:**
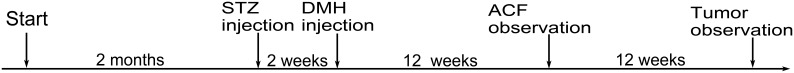
Time course of the experiment and the animal treatment procedures. STZ was administered as 35 mg/kg, i.p. and DMH was administered as 25 mg/kg/week, i.p. The abbreviations denote STZ: Streptozotocin; DMH: 1,2-dimethylhydrazine; ACF: Aberrant crypt foci.

The time course of the experiment is shown in Figure 1. One week after the beginning of the experiment, one rat in the control group died unexpectedly. Due to a severe weight loss, one rat in the STZ+DMH group also died 3 weeks before tumor observation.

**Figure 2:**
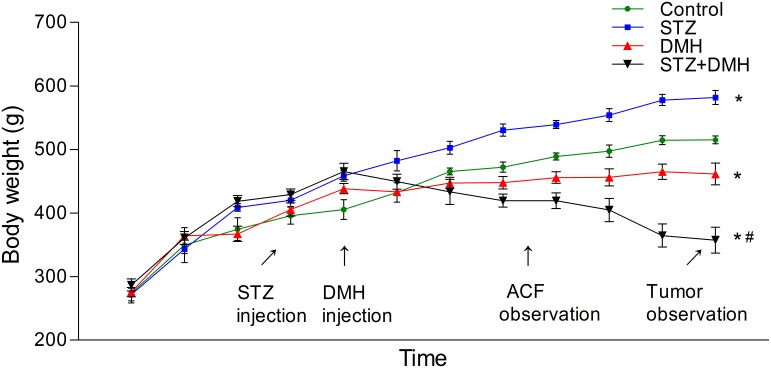
Body weight of rats throughout the entire experiment. Values are expressed as mean ± S.E.M. and analyzed using unpaired student’s T test at P<0.05. At the conclusion of the experiment, *P<0.05 vs. Control group. ^#^P<0.05 vs. DMH group.

Figure 2 illustrates that the body weight of all rats kept increasing over the two-month protocol, and feeding of HFD resulted in a statistically significant increase in body weight compared to NPD-fed rats (413.68±2.76 vs. 370.90±4.69, P<0.05). Following the injection of STZ, the body weight of rats in the control group and STZ group kept increasing during the entire duration of the experiment, and the weight of those in the STZ group increased faster than those in the control group. However, along with the injection of DMH, the body weight of rats began to decrease and the weight of those in the STZ+DMH group decreased faster than those in DMH group. At the end of the experiment, the body weight of rats in the STZ group was increased significantly compared to the control group (581.82±4.88 vs. 515.07±2.76, P<0.05). In contrast, DMH-induced (DMH group and STZ+DMH group) weights were decreased compared to those of the control group (461.25±7.68 or 357.24±9.14 vs. 515.07±2.76, P<0.05), and the weights of rats in the STZ+DMH group were lower than those in the DMH group (357.24±9.14 vs. 461.25±7.68, P<0.05).

### Serological analysis

Blood biochemical indices (FBG, TG, TC, HDL-C, LDL-C, INS) were measured immediately before and one week after the injection of vehicle or STZ (Table 2). Serum FBG levels as well as TG, LDL-C and INS were all significantly increased in HFD-fed rats compared with NPD-fed rats before the injection of STZ (P<0.05, respectively). However, there was a reduction of HDL-C (0.41±0.02 vs. 0.51±0.03, P<0.05). Injection of STZ resulted in a significant increase of FBG, TG, TC and LDL-C associated with a significant reduction of HDL-C. In addition, although the injection of STZ produced a reduction of INS level in HFD-fed rats (72.86±3.75 vs. 89.72±2.62, P<0.05), the level of INS was still considerably higher than that of in NPD-fed rats (72.86±3.75 vs. 30.90±3.28, P<0.05).

**Table 2:** Level of blood biochemical indexs.

	NPD-fed		HFD-fed	
	Before	After	Before	After
FBG (mmol/L)	4.43±0.07	4.60±0.08	5.34±0.11*	14.53±0.29** #
TG (mmol/L)	0.61±0.04	0.59±0.04	1.15±0.07*	6.75±0.25** #
TC (mmol/L)	1.17±0.04	1.13±0.04	1.40±0.04	4.07±0.26** #
HDL-C (mmol/L)	0.51±0.03	0.49±0.02	0.41±0.02*	0.22±0.02** #
LDL-C (mmol/L)	0.77±0.07	0.78±0.04	0.95±0.06*	1.41±0.07** #
INS (µIU/ml)	29.19±2.91	30.90±3.28	89.72±2.62**	72.86±3.75** #

Blood samples were drawn immediately before and 1 week after injection of STZ. NPD-fed indicate Control group and DMH group. HFD-fed indicate STZ group and STZ+DMH group. Values are expressed as mean ± S.E.M. Unpaired student’s T test at P<0.05 was used to analyze the difference between the NPD-fed and HFD-fed rats. The abbreviations denote FBG: fasting blood glucose, TG: triglyceride, TC: total cholesterol, HDL-C: high-density lipoprotein cholesterol, LDL-C: low-density lipoprotein cholesterol and INS: insulin.

*P<0.05 vs. Before of NPD groups.

**P<0.01 vs. After of NDP groups.

# P<0.01 vs. Before of HFD groups.

### ACF observation

**Figure 3:**
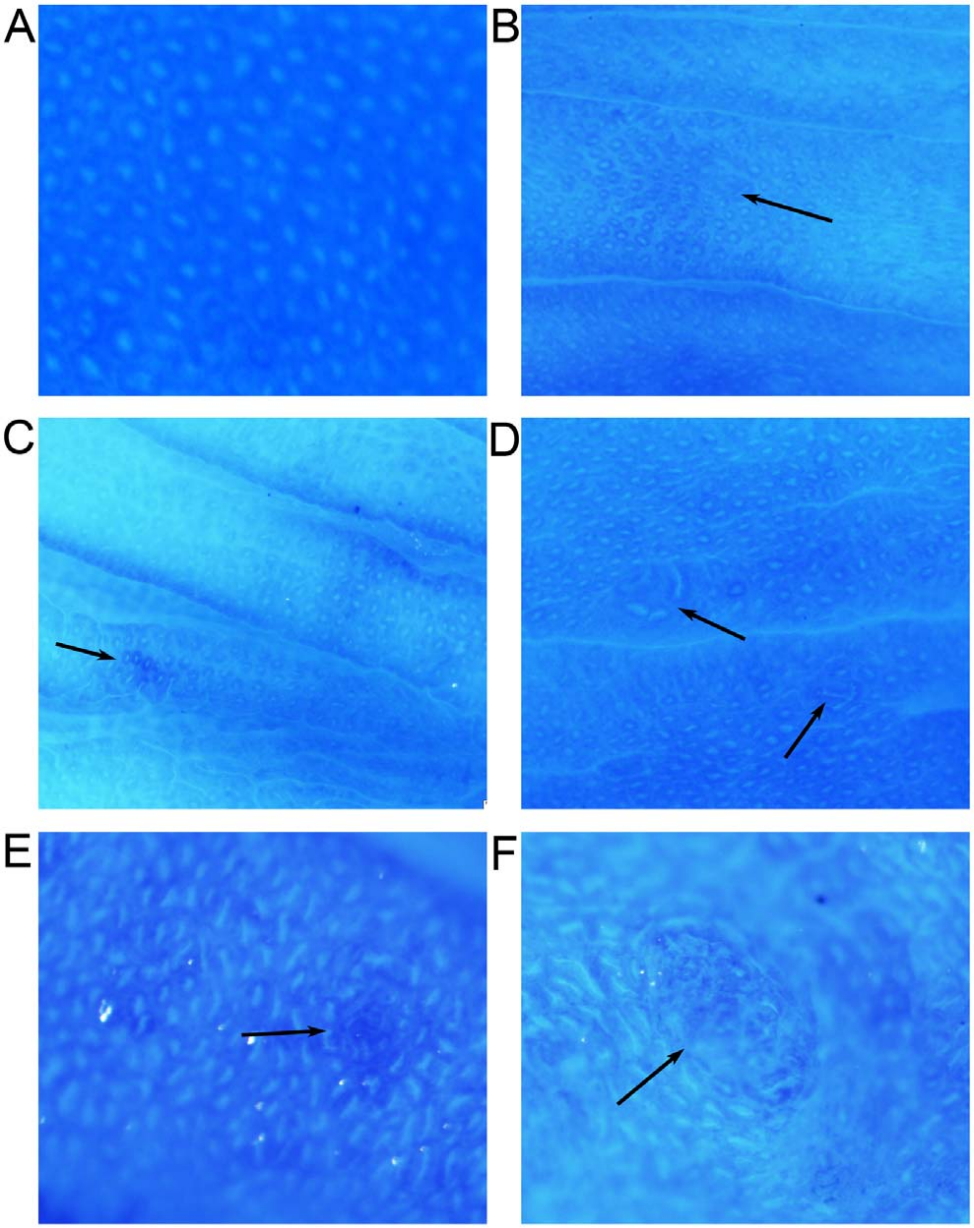
Formation of ACF induced by DMH in the rat colon. Fig. 3A: normal crypt foci (100×). Fig. 3B: Crypt enlargement and deformation (40×). Fig. 3C: ACF formed by 2 aberrant crypts (40×). Fig. 3D: ACF formed by 3 aberrant crypts (40×). Fig. 3E: ACF formed by ≥3 aberrant crypts (40×). Fig. 3F: tumor-like tissue (40×).

After 12 weeks of DMH injection, colonic ACF were identified in the DMH group and STZ+DMH group (Figure 3) but not in the control group and STZ group. Unpaired student’s T test was used to detect the difference between DMH group and STZ+DMH group. As shown in Table 3, the total number of ACF in the STZ+DMH group was significantly higher than that in the DMH group (230.80±8.85 vs. 141.00±5.26, P<0.05). The number of foci containing 1 crypt, 2 crypts and ≥3 crypts were also significantly increased. In the STZ+DMH group, the number of foci containing more than 3 crypts was even higher than that of foci containing 2 crypts (74.60±3.11 vs. 45.40±1.57, P<0.05). In addition, some tissues that resemble tumor were found in the STZ+DMH group (Figure 3F).

**Table 3:** DMH-induced ACF in rat colon.

Group	n	Total Numberof ACF/colon	Number of foci containing		
			1 crypt	2 crypts	≥3 crypts
Control	5	0	0	0	0
STZ	5	0	0	0	0
DMH	5	141.00±5.26	80.60±3.23	45.40±1.57	15.00±1.30
STZ+DMH	5	230.80±8.85*	99.00±4.66*	57.20±2.69*	74.60±3.11* #

Five rats from each group were sacrificed for ACF identification. 1 crypt indicate ACF formed by 1 aberrant crypt, 2 crypt indicate ACF formed by 2 aberrant crypt, ≥3 crypt indicate ACF formed by at least 3 aberrant crypt. Values are expressed as mean ± S.E.M. and analyzed using unpaired student’s T test at P<0.05.

*P<0.05 vs. DMH group.

# P<0.05 vs. the number of foci containing 2 crypt in STZ+DMH group.

### Incidence, number and volume of colonic tumors

**Figure 4:**
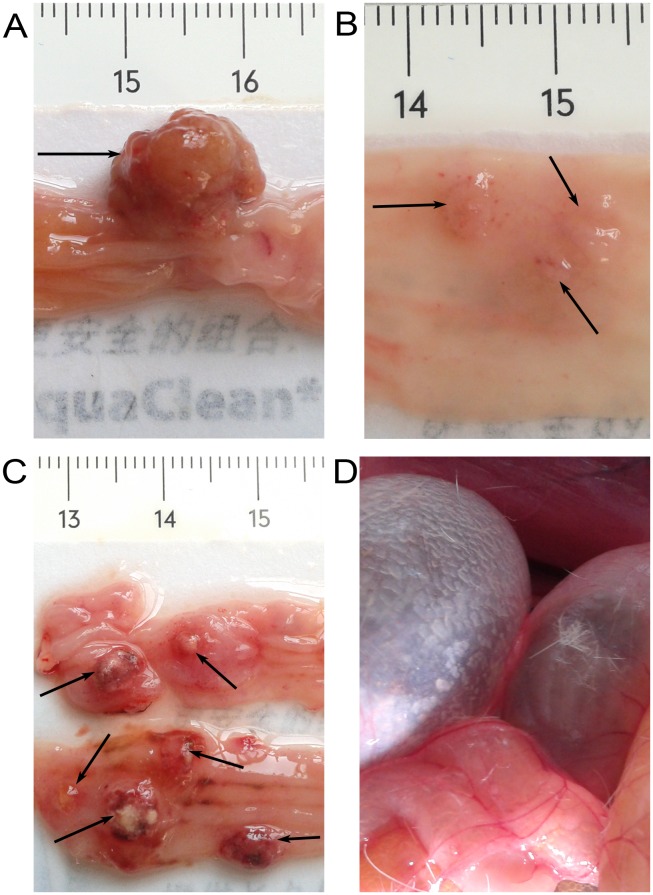
Tumor tissues obtained from DMH-induced rats. Fig. 4A: The largest tumor obtained from the STZ+DMH group. Fig. 4B: Several small tumors together isolated from the DMH group. Fig. 4C: Several tumors collected from one rat in the STZ+DMH group. Fig. 4D: Tumor growth in the intestinal lumen interfering with bowel movements, resulting in difficult defecation and building of gas in the intestine in some rats.

At the conclusion of the experiment, all rats were sacrificed (10 in the DMH group and 9 in the STZ+DMH group). Seven rats in the DMH group and eight in the STZ+DMH group were found to have tumors (Table 4). The incidence of tumors in the STZ+DMH group was higher than that in the DMH group, but the difference did not reached statistical significance (88.89% vs. 70.00%, P>0.05). The volume of tumors, total number of tumors and average number of tumors in the STZ+DMH group were significantly increased compared with that in the DMH group (P<0.05, respectively). The volume of the biggest tumor found in the STZ+DMH group reached to 665.5 mm^3^, but the smallest in that group was only 6 mm^3^. In contrast, the volume of the biggest tumor in the DMH group was only 75 mm^3^, and the smallest was only 4 mm^3^ (Figure 4). The tumors growth in the intestinal lumen interfered with bowel movements, resulting in difficult defecation and building of intestinal gas in some rats (Figure 4D).

**Table 4:** Incidence, number and volume of tumors.

	Group	
	DMH	STZ+DMH
n	10	9
Tumor-bearing rats	7	8
Tumor Incidence (%)	70.00%	88.89%
Number of tumors	16	30
Average tumor number	1.6±0.45	3.33±0.53*
Average tumor number oftumor-bearing rats	2.28±0.42	3.75±0.37*
Volume of Tumors (mm^3^)	24.09±4.40	89.02±23.87*

All rats were sacrificed at the conclusion of the experiment for tumor observation. Tumor-bearing rats indicate that the tumors were visualized in general by gross examination in colon tissue. Values are expressed as mean ± S.E.M. The parameters of tumor incidence was analyzed using Chi square test at P<0.05. The average tumor number, average tumor number of tumor-bearing rats and volume of tumors were analyzed using unpaired student’s T test at P<0.05.

*P<0.05 vs. DMH group.

### Proliferation index of colorectal tumor cells

**Figure 5:**
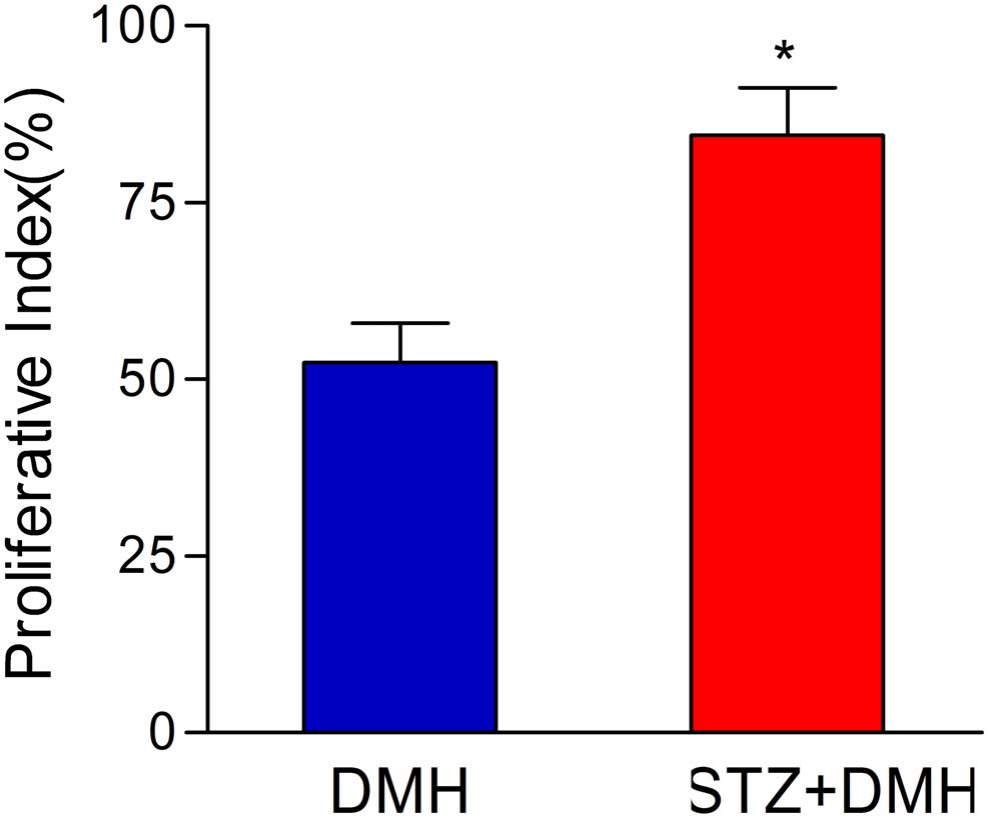
A bar chart demonstrating the proliferation index (PI) of colorectal tumor cells in DMH-induced colon tissues (DMH group and STZ+DMH group). The PI was expressed as the percentage of PCNA-positive nuclei among the total number of cells counted. Values are expressed as mean ± S.E.M. and analyzed using unpaired student’s T test at P<0.05. *P<0.05 vs. DMH group.

PCNA was strongly expressed in DMH-induced colon tumor cells, especially in the DMH+STZ group. As shown in Figure 5, the proliferation index (PI) in the DMH+STZ group was significantly higher than that in DMH group (84.5±6.72 vs. 52.3±5.58, P<0.05). These data indicated that the DMH treatment significantly promoted cell proliferation, which was accelerated by STZ-induced diabetes.

### Analysis of glycolytic enzymes

**Figure 6:**
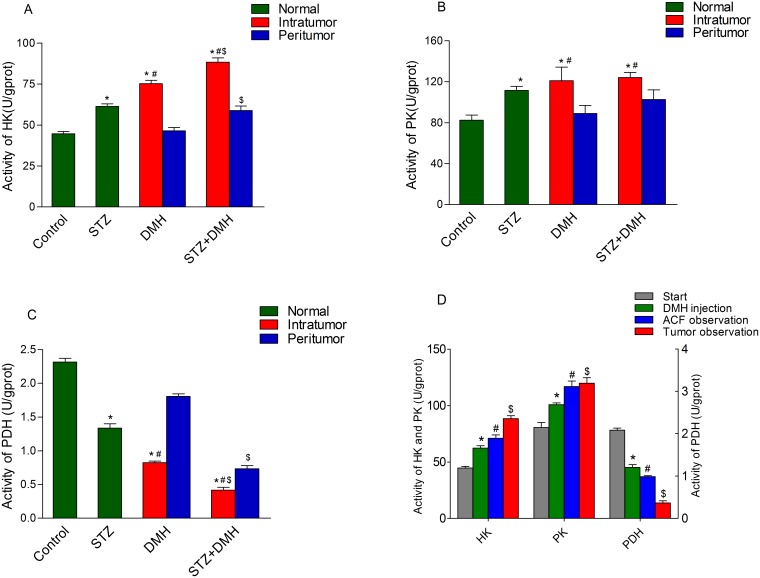
Bar charts demonstrating the enzyme activities in colon tissues. No tumor formation was detected in the Control group and STZ group, so dissect the samples at random. Samples were collected from the intratumoral and the peritumoral regions respectively in the DMH group and STZ+DMH group. The activities of hexokinase (HK), pyruvate kinase (PK) and pyruvate dehydrogenase (PDH) in colonic tissues were measured. Values are expressed as mean ± S.E.M. and analyzed using unpaired student’s T test at P<0.05. Fig. 6A: Analysis of HK. *P<0.05 vs. Control group. ^#^P<0.05 vs. Peritumor respectively. ^$^P<0.05 vs. DMH group respectively; Fig. 6B: Analysis of PK. *P<0.05 vs. Control group. ^#^P<0.05 vs. Peritumor respectively; Fig. 6C: Analysis of PDH. *P<0.05 vs. Control group. ^#^P<0.05 vs. Peritumor respectively. ^$^P<0.05 vs. DMH group respectively. Fig. 6D: Analysis of HK, PK and PDH activities at different time course in the STZ+DMH group. *P<0.05 vs. Start point respectively. ^#^P<0.05 vs. STZ injection respectively.

No tumor formation was detected in the control group and STZ group, therefore these colonic tissue samples were dissected at random. In the DMH group and STZ+DMH group, colonic samples were collected from the peritumoral and the intratumoral regions respectively. The activities of HK, PK and PDH in colonic tissues were measured and shown in Figure 6.

As shown in Figure 6A, the activities of HK in the intratumoral tissues in the DMH group and STZ+DMH group were significantly increased compared with that in peritumoral tissues and the normal tissues (including the control group and STZ group, P<0.05, respectively). The activity of HK in the STZ group was also higher than that in the control group and the peritumoral tissues in the DMH group (61.26±1.57 vs. 44.61±1.48 and 48.01±1.70, P<0.05, respectively). Regardless of the intratumoral or peritumoral location, the HK activity in the STZ+DMH group was higher than that in the DMH group (P<0.05, respectively).

The activities of PK (Figure 6B) in the intratumoral tissues of the colon in the DMH group and STZ+DMH group were all significantly increased compared with that in the peritumoral tissues and were also higher than the control group (P<0.05, respectively). However, no difference in the intratumoral tissues was found between DMH group and STZ+DMH group. The activity of PK in the STZ group was higher than that in the control group (111.42±4.10 vs. 82.40±4.96, P<0.05) and was higher than the peritumoral tissues in the STZ+DMH group without reaching statistical significance (P>0.05). The activity of PK in the peritumoral tissues in the STZ+DMH group was higher than the control group (102.66±4.68 vs. 82.40±4.96, P<0.05), but no difference was observed between the control group and the peritumoral tissues in the DMH group.

A significant reduction of PDH activity (Figure 6C) was found in diabetic rats when compared with the control group (1.34±0.05 vs. 2.32±0.05, P<0.05). In addition, the activity of PDH in the intratumoral tissue was considerably decreased when compared with the peritumoral tissues (P<0.05, respectively). Both in the introtumoral and peritumoral tissues, the activity of PDH in the STZ+DMH group were significantly lower than the DMH group (P<0.05, respectively).

Four points in time course of the experiment were selected to indicate the changes of these three enzymes activity in the STZ+DMH group (Figure 6D). No rats were sacrificed at the start point and DMH injection point, and the data at these two points were replaced by the data in control group and STZ group at the end of the experiment respectively. As shown in Figure 6D, the activities of HK and PK were all significantly increased after the type 2 diabetes were induced (62.26±2.06 vs. 44.61±1.48, 100.79±1.49 vs. 80.54±4.27, P<0.05). Whereas, there was a reduction in the activity of PDH (1.34±0.05 vs. 2.32±0.05, P<0.05). Moreover, the current results showed that repeated DMH injection resulted in a further increase in HK and PK and reduction in PDH activities (P<0.05, respectively).

## Discussion

One of the main objectives of the present work was to determine whether type 2 diabetes mellitus could increase the risk of colorectal cancer in an animal model. The second objective was to investigate the possible mechanisms involved in this process. Therefore, the activities of HK, PK and PDH in colorectal tissues were measured to evaluate the contribution of metabolic changes in the process of tumorigenesis. Thus, our initial attempts were directed towards generating a model of type 2 diabetes that would closely reflect the natural history and metabolic characteristics of human type 2 diabetes, after which colorectal cancer was induced using DMH.

The animal model of type 2 diabetes was induced using high-fat feeding in combination with a low dose of STZ (35 mg/kg). Mansor et al. have reported that this model could not only replicate the pathology of human diabetes but also mimic the disease process, but the dose of STZ must be carefully chosen [22]. Diabetic rats induced by different doses of STZ (15, 25, 35, 45 and 55 mg/kg) combined with HFD were studied by K. Srinivasan et al. [23]. The metabolic changes induced by higher doses of STZ (45 and 55 mg/kg) resembled the type 1 diabetes phenotype. In contrast, low doses of STZ (15 and 25 mg/kg) did not produce significant hyperglycemia. Hence, HFD in combination with a low dose of STZ (35 mg/kg) was the most desirable method of inducing type 2 diabetes. In our study, after 2 months of dietary manipulation, the HFD-fed rats were already mildly hyperglycemic and the body weight increased rapidly due to the consumption of a diet rich in energy in the form of saturated fats compared to NPD-fed rats. Meanwhile, the level of INS increased considerably compared to the NPD-fed groups. These data indicated that the HFD-fed rats had already raised insulin resistance with compensatory hyperinsulinemia [25]. After the injection of STZ, the level of the blood biochemical indices in the HFD-fed groups were all significantly increased, except for HDL-C and INS. The reduction of INS may due to destruction of pancreatic beta cells by injection of STZ [26],[27], but the level of INS was still higher than that of the NPD-fed rats. As Srinivasan demonstrated, the elevated concentrations of glucose were relatively stable in this model, which could be used for long-term studies on diabetic complications [23].

In the present study, the DMH-induced colon carcinogenesis model was used for evaluation of the risk of cancer in diabetic rats in light of the formation of ACF and tumor incidence. Mohania D et al, successfully developed a colorectal cancer model with this agent [28]. ACF was selected as an intermediate biological evaluation index in the pathogenesis of colorectal cancer. ACF refer to the abnormal change of normal foci. Greaten of foci, epithelium thickening, and foci with several or multiple mutations gathered together in a focal distribution are characteristics of ACF [29]–[32]. Both in rodents or humans, in the pathogenesis of colorectal cancer induced by carcinogens, ACF are precancerous lesions of colorectal cancer and are considered to be good biological markers for evaluating the effect of drugs that are used to prevent and control the formation of colorectal cancer in rats [32],[33]. Rodrigues et al. demonstrated that the DMH-induced colorectal cancer model in rats is a valid tool to investigate the association of ACF with colorectal cancer. ACF may be regarded as early morphological markers in the pathogenesis of well-differentiated tumors in colon carcinogenesis [32]. The results of this study indicated that the number of ACF and the number of foci containing different number of crypts in the STZ+DMH group increased compared with the DMH group. In the DMH group, 89.4% ACF had one or two crypts, and only 10.6% ACF had three or more crypts. However, in the STZ+DMH group, the incidence of ACF with one or two crypts was 67.7% and 32.3% had three or more crypts. In contrast, the STZ group had no ACF formation. Epidemiologic findings have shown that the incidence of colorectal cancer in diabetic patients was closely related to the diabetes duration. Thus, we inferred that a much longer time is needed for the rats in the STZ group to develop ACF. The significant increase of foci containing ≥3 crypts and the detection of tumor-like tissue in the STZ+DMH group indicated that the presence of diabetes mellitus shortened the duration of tumorigenesis. In accordance, Zaafar et al. have reported that diabetes promotes the size of cell and the inflammatory reactions in the mucosal layer like lymphoid proliferation, congestion of blood vessels and fibrosis [34]. At the end of our present study, tumors were found both in the DMH group and STZ+DMH group. The incidence, number and size of tumors in the STZ+DMH group increased compared with that of DMH group. In contrast, no tumors were found in the STZ group. Whereas, no lesions in general were visualized by gross examination in Zaafar’s study [34]. Different animals were selected in the experiments may be the possible explanation for this phenomenon. Mice were used in their experiment while rats were selected in our study. The gap of body size may reflect the size of the tumor, so tumors were difficult to be visualized in mice colon tissues. However, there was no significant difference in the incidence of tumors between the STZ+DMH group and DMH group in current result. Possible explanations for this phenomenon may include the limited sample size, with only 15 rats in each group. Overall, these data demonstrated that diabetic rats have a high risk of colorectal cancer.

Rapid proliferation is the main feature of tumor cells. Proliferating cell nuclear antigen (PCNA) is a DNA clamp that acts as a processivity factor for DNA polymerase δ in eukaryotic cells and is essential for replication. Bostick et al. have reported that the expression of PCNA is closely related to the proliferation of cell in colon tissue and can be used as a very reliable indicator to evaluate the proliferation dynamics of tumor cell [35]. The present study demonstrated that PCNA was highly expressed in all tumor tissues and that the expression of PCNA was increased in colonic ACF compared with that of normal intestinal glands. PCNA expression can reflect cellular proliferation activity and is used as a reliable index for evaluating the kinetics of tumor cell proliferation. Our results showed that PCNA was expressed in DMH-induced colon cancer cells, and the strongest proliferation activity was observed in the DMH+STZ group. These data indicated that STZ treatment could promote DMH-induced cell proliferation.

Previous hypothesis about the possible mechanisms involved in DM-related cancer was the abnormal increase of insulin-like growth factor-1 (IGF-1) and vascular endothelial growth factor (VEGF) in the serum that can lead to the occurrence of tumors by promoting the transformation and proliferation of colorectal epithelial cells, influencing the cell cycle and inhibiting cell apoptosis. As shown in Zaafar’s report [34], the level of IGF-1 and VEGF were all increased in diabetic mice and diabetic mice had higher susceptibility to DMH-induced colon cancer compared to nondiabetics. In current study, the hypothesis about the possible mechanisms involved in DM-related cancer was focused on the changes of glycolytic enzymes’ activity. Recent studies have shown that several molecules involved in the Warburg effect may be rational targets for cancer therapy [16],[17]. All functions in cells are energy-dependent. The oxidation of glucose through mitochondrial oxidative phosphorylation can produce 30–32 molecules of net ATP per molecule of glucose, but glycolysis generates only two molecules of ATP. HK and PK are key enzymes in glycolysis. Tumor cells exhibit a high rate of glycolysis under aerobic conditions, a phenomenon known as the Warburg effect. It had been reported that insulin could induce HK gene transcription, and this, in turn, increased glucose phosphorylation in these cells [36]. A study by Mansor showed that the activity of PDH, a highly regulated enzyme of mitochondrial glucose metabolism, was significantly decreased in the diabetic model [22]. PDH is also negatively regulated by PDK via phosphorylation, and the level of PDK has been shown to be significantly increased in diabetic models [22],[37]. The phosphorylation of PDH can prevent the movement of pyruvate into the mitochondrial matrix [37],[38]. The results of current study showed that the activities of HK and PK significantly increased in diabetic rats, whereas the activity of PDH was reduced. In addition, the activities of PK and HK in tumoral tissues were significant higher than that in peritumoral tissues. The imbalance of carbohydrate metabolism between glycolysis and oxidative phosphorylation could result in the generation of lactic acid and hydrogen ions. The acidic microenvironment provides a proliferative advantage for tumorigenesis. In addition, many intermediate products in glycolysis can be exploited by tumor cells to synthesize proteins, nucleic acids and lipids, thereby providing the necessary materials for the growth and proliferation of tumor cells [39]. These data indicated that the increasing activity of glycolytic enzymes in diabetes might play a role in the occurrence of colorectal cancer. However, the detailed mechanism needs further investigation.

In conclusion, our findings demonstrated that type 2 diabetes mellitus is a high risk factor for colorectal carcinogenesis. The mechanism for this risk may involve the dysregulation of glucose metabolism in diabetes. Hyperglycemia and hyperinsulinemia in diabetic mellitus might impact the activity of enzymes catalyzing the biochemical reactions of carbohydrate metabolism. The high rate of glycolysis in colon tissues provided a proliferative advantage for tumorigenesis. This study indicated several rational targets for the prevention of diabetic complications and tumor therapy.
